# Insulin rapidly stimulates l-arginine transport in human aortic endothelial cells via Akt

**DOI:** 10.1016/j.bbrc.2011.08.048

**Published:** 2011-09-09

**Authors:** Christine F. Kohlhaas, Valerie A. Morrow, Neelam Jhakra, Vrushali Patil, John M.C. Connell, John R. Petrie, Ian P. Salt

**Affiliations:** Institute of Cardiovascular and Medical Sciences, College of Medical, Veterinary and Life Sciences, University of Glasgow, Davidson Building, Glasgow G12 8QQ, United Kingdom

**Keywords:** Ad.control, control adenovirus, Ad.Akt-CA, adenovirus expressing constitutively active mutant Akt, Ad.Akt-DN, adenovirus expressing dominant negative mutant Akt, BAEC, bovine aortic endothelial cell, eNOS, endothelial nitric oxide synthase, HAEC, human aortic endothelial cell, Hsp90, heat shock protein 90, HUVEC, human umbilical vein endothelial cell, MARCKS, myristoylated alanine-rich protein kinase C substrate, NO, nitric oxide, PI3 K, phosphatidylinositol 3’kinase, PKC, protein kinase C, PMA, phorbol 12-myristate 13-acetate, Insulin, Nitric oxide, Arginine, Transport, Endothelium

## Abstract

Insulin stimulates endothelial NO synthesis, at least in part mediated by phosphorylation and activation of endothelial NO synthase at Ser1177 and Ser615 by Akt. We have previously demonstrated that insulin-stimulated NO synthesis is inhibited under high culture glucose conditions, without altering Ca^2+^-stimulated NO synthesis or insulin-stimulated phosphorylation of eNOS. This indicates that stimulation of endothelial NO synthase phosphorylation may be required, yet not sufficient, for insulin-stimulated nitric oxide synthesis. In the current study we investigated the role of supply of the eNOS substrate, l-arginine as a candidate parallel mechanism underlying insulin-stimulated NO synthesis in cultured human aortic endothelial cells. Insulin rapidly stimulated l-arginine transport, an effect abrogated by incubation with inhibitors of phosphatidylinositol-3′-kinase or infection with adenoviruses expressing a dominant negative mutant Akt. Furthermore, supplementation of endothelial cells with extracellular l-arginine enhanced insulin-stimulated NO synthesis, an effect reversed by co-incubation with the l-arginine transport inhibitor, l-lysine. Basal l-arginine transport was significantly increased under high glucose culture conditions, yet insulin-stimulated l-arginine transport remained unaltered. The increase in l-arginine transport elicited by high glucose was independent of the expression of the cationic amino acid transporters, hCAT1 and hCAT2 and not associated with any changes in the activity of ERK1/2, Akt or protein kinase C (PKC). We propose that rapid stimulation of L-arginine transport contributes to insulin-stimulated NO synthesis in human endothelial cells, yet attenuation of this is unlikely to underlie the inhibition of insulin-stimulated NO synthesis under high glucose conditions.

## Highlights

► Regulation of NO synthesis by l-arginine transport in endothelial cells. ► L-arginine transport stimulated by insulin and high glucose. ► Insulin-stimulated l-arginine transport dependent on Akt activation. ► Culture glucose concentrations have no effect on l-arginine transporter levels.

## 1 Introduction

Cardiovascular disease is the principal cause of morbidity and mortality in patients with diabetes and is associated with endothelial dysfunction, characterised by reduced nitric oxide (NO) bioavailability, a key early mechanism in the progression of atherosclerosis [1]. The molecular mechanisms that underlie the association between diabetes, atherosclerosis and endothelial dysfunction are not, however, fully characterised. Insulin is a direct-acting vasodilator in intact vessels [2,3] and stimulates NO production in cultured endothelial cells [4,5]. We and others have reported that insulin-stimulated NO synthesis is the result of phosphatidylinositol-3′-kinase (PI3 K)-mediated stimulation of Akt (also known as protein kinase B (PKB)) and subsequent phosphorylation and activation of endothelial NO synthase (eNOS) at Ser1177 and Ser615 [5–7].

We have previously demonstrated that culture of human aortic endothelial cells (HAECs) in high glucose abrogated insulin-stimulated NO synthesis, without altering Ca^2+^-stimulated NO synthesis, indicating a specific downregulation of insulin signalling by high glucose [5]. Furthermore, high glucose had no effect on insulin-stimulated phosphorylation of eNOS at Ser1177 or Ser615 [5,7]. Similar studies in bovine aortic endothelial cells (BAECs) also demonstrated an inhibition of insulin-stimulated NO synthesis by culture in high glucose, with only a modest inhibition of insulin-stimulated Ser1179 (bovine equivalent of Ser1177) phosphorylation [8]. We therefore reasoned that the dissociation of eNOS phosphorylation and NO synthesis specific to insulin-stimulated cells implies that stimulation of Akt and subsequent phosphorylation of eNOS may be required, yet insufficient, for insulin-stimulated NO synthesis in cells cultured in high glucose. Candidate mechanisms that might underlie this eNOS phosphorylation-independent mechanism, include altered supply of the eNOS substrate, l-arginine [9], or altered regulation of eNOS activity by interactions with proteins including heat shock protein (Hsp) 90 [10] and caveolin-1 [11].

Long-term (8 h) stimulation with insulin has previously been demonstrated to increase the transport of l-arginine in cultured human umbilical vein endothelial cells (HUVECs), in a manner sensitive to the protein synthesis inhibitor, cycloheximide [9]. It has also been reported that l-arginine transport can be rapidly increased by various stimuli in HUVECs, indicating a capacity for regulation of transport independent of changes in transporter protein expression [12,13]. In endothelial cells, l-arginine transport is mediated by system y^+^, reflecting activity of the cationic amino acid (CAT) transporters and system y^+^L, which reflects heterotrimeric amino acid transporter activity [14]. The potential rapid effects of insulin on l-arginine transport, and thereby eNOS substrate availability have yet to be characterised. In the current study we investigated the role of l-arginine transport as a potential mechanism underlying insulin-stimulated NO synthesis.

## 2 Materials and methods

### 2.1 Materials

Cryopreserved HAECs were purchased from Promocell (Heidelberg, Germany) and large vessel endothelial cell media obtained from TCS Cellworks (Botolph Claydon, Buckinghamshire, UK). L-[2,3,4,5-^3^H] arginine was obtained from GE Healthcare (Little Chalfont, Buckinghamshire, UK). l-lysine was obtained from Sigma (Poole, Dorset, UK). PD98059, LY294002 and phorbol 12-myristate 13-acetate (PMA) were obtained from Merck (Nottingham, UK). Rabbit anti-phospho-myristoylated alanine-rich protein kinase C substrate (MARCKS) (Ser152/156), anti-phospho-extracellular signal regulated kinase (ERK)1/2 (Thr202/Tyr204); anti-ERK1/2; anti-Akt and anti-phospho-Akt (Ser473) antibodies were from New England Biolabs UK (Hitchin, UK). Rabbit anti-hCAT1 antibodies were from Proteintech Europe (Manchester, UK). Rabbit anti-hCAT2 antibodies were from Aviva Systems Biology (San Diego, USA). Mouse anti-glyceraldehyde-3-phosphate dehydrogenase (GAPDH) antibodies were from Ambion (Huntingdon, UK). Replication-defective adenovirus vectors expressing mouse Akt were a kind gift from Dr. Kenneth Walsh, Boston University School of Medicine, Boston, USA and have been described previously [7]. All other reagents were from sources described previously [5,7].

### 2.2 Cell culture

HAECs were grown in large vessel endothelial cell medium containing 5 mM glucose and passaged when at 80% confluence as described previously [5,7]. Cells were used for experiments between passages 3 and 6 when 70–80% confluent. Cells subjected to high glucose were supplemented with glucose to obtain a final concentration of 25 mM or mannitol as an osmotic control as described previously [5,7].

### 2.3 Preparation of adenoviruses and infection of HAECs

Adenoviruses expressing dominant negative Akt (Ad.Akt-DN), constitutively active Akt (Ad.Akt-CA) or control adenoviruses (Ad.control) were propagated and purified as described previously [7]. HAECs were infected with 5 pfu/cell adenovirus in complete medium and the cells cultured for 48 h prior to experimentation, with the final 24 h in serum-free medium. Under these conditions after infection with a green fluorescent protein (GFP)-expressing virus, the majority (>95%) of HAECs expressed GFP [7].

### 2.4 l-Arginine transport assay

Cells cultured in 6-well plates were preincubated in Krebs Ringer HEPES (KRH) buffer (119 mM NaCl, 4.75 mM KCl, 1.2 mM MgSO_4_, 5 mM NaHCO_3_, 1.3 mM CaCl_2_, 20 mM HEPES pH7.4, KH_2_PO_4_, 5 mM glucose) supplemented with a further 20 mM (25 mM final) glucose or mannitol at 37 °C for 2 h and subsequently reagents (insulin, wortmannin, LY294002, PD98059 or bisindolylmaleimide I) added for the durations described. Plates were transferred to preheated hot plates at 37 °C for the assay. The transport assay was initiated by the addition of 80 μM [^3^H]L-arginine (0.19 MBq/ml) and after incubation for 1 min, transport was stopped by plunging the plates into ice-cold PBS. Plates were air-dried prior to lysis of the cells with 1% (v/v) Triton-X100. Cell-associated ^3^H was measured by liquid scintillation counting and specific transport calculated by subtracting values obtained from control plates incubated under identical conditions that had 10 mM l-lysine added 1 min prior to assay. l-Arginine transport was normalised to total cellular protein within each well.

### 2.5 Evaluation of HAEC NO synthesis

HAECs cultured in 12-well plates were incubated in serum-free medium overnight. Cells were preincubated for 1 h at 37 °C in 0.5 ml/well KRH buffer. The medium was removed and replaced with fresh KRH buffer (0.5 ml/well) in the presence of various concentrations of test substances. After incubation for various durations, aliquots of media were removed and analysed using a Sievers 280A NO analyzer as described previously [5,7]. The appropriate control experiments were performed in the presence of the eNOS inhibitor, L-NAME (0.5 mM). Data is presented as L-NAME-sensitive NO synthesis.

### 2.6 Preparation of cell lysates, SDS PAGE and Western blotting

Cell lysates were prepared, proteins resolved by SDS–PAGE and subjected to immunoblotting with the antibodies indicated as described previously [5,7].

### 2.7 Statistics

Unless stated otherwise, results are expressed as the mean ± SEM. Statistically significant differences were determined using a two-tailed Student’s *t* test or ANOVA as appropriate, with *p *< 0.05 deemed significant.

## 3 Results

**Fig. 1:**
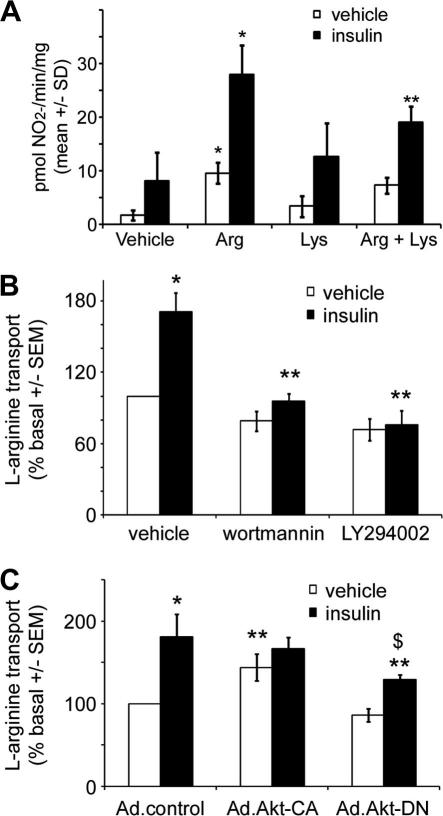
Regulation of NO synthesis by l-arginine and l-arginine transport by insulin in HAECs. (A) Basal and insulin-stimulated (1 μM, 5 min) NO synthesis was assessed in HAECs pre-incubated in l-arginine (80 μM, 5 min) and/or l-lysine (10 mM, 5 min). Results are shown from six independent experiments. ^∗^p < 0.05 relative to value in absence of l-arginine, ^∗∗^p < 0.05 relative to value in absence of lysine. The rate of l-arginine transport was assessed after (B) incubation of HAECs in the presence or absence of insulin (1 μM, 10 min), wortmannin (100 nM, 45 min) or LY294002 (10 μM, 30 min). (C) HAECs were infected with the indicated adenoviruses for 48 h, prior to incubation in the presence or absence of insulin (1 μM, 10 min). Results are shown from (B) six or (C) three independent experiments. ^∗^*p* < 0.01 relative to value in absence of insulin, ^$^*p* < 0.05 relative to value in absence of insulin. ^∗∗^*p* < 0.05 relative to value in absence of drug or control adenoviruses.

We first addressed whether l-arginine supplementation affects insulin-stimulated NO synthesis. In the presence of physiological concentrations of extracellular l-arginine (80 μM), insulin-stimulated NO synthesis was significantly increased approximately 3.5-fold, an effect attenuated by the addition of 10 mM l-lysine, an inhibitor of l-arginine transport (Fig. 1A).

Insulin rapidly (10 min) significantly stimulated the transport of l-arginine in HAECs 1.7 ± 0.2-fold (Fig. 1B). Preincubation with the PI3 K inhibitor, wortmannin, completely and significantly inhibited insulin-stimulated l-arginine transport in HAECs (Fig. 1B). Preincubation of HAECs with the structurally unrelated PI3 K inhibitor, LY294002 gave quantitatively similar results to those with wortmannin (Fig. 1B).

Next we investigated the role of the downstream PI3 K effector, Akt, in insulin-stimulated l-arginine transport using Ad.Akt-DN or Ad.Akt-CA. Infection of HAECs with Ad.Akt-DN significantly reduced insulin-stimulated l-arginine transport by approximately 30% without reducing basal transport (Fig. 1C). In HAECs infected with Ad.Akt-CA, basal l-arginine transport was significantly increased by 44% compared to control adenovirus infected HAECs, yet insulin stimulation had no further significant effect on l-arginine transport (Fig. 1C).

**Fig. 2:**
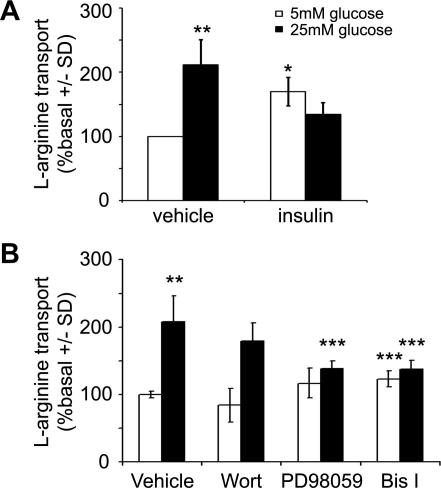
The effect of high glucose on l-arginine transport in HAECs. The rate of l-arginine transport was assessed (A) in HAECs after incubation in the glucose concentrations indicated for 48 h and subsequent incubation in the presence or absence of insulin (1 μM, 10 min) or (B) cultured in 5 mM or 25 mM glucose prior to incubation in the presence or absence of wortmannin (wort; 100 nM, 40 min), PD98059 (10 μM, 30 min) or Bisindolylmaleimide I (Bis I; 100 nM, 30 min). Results are shown from (A) six or (B) five independent experiments. ^∗^*p* < 0.005 relative to value in absence of insulin, ^∗∗^*p* < 0.05 relative to value in presence of 5 mM glucose, ^∗∗∗^*p* < 0.05 relative to value in vehicle.

**Fig. 3:**
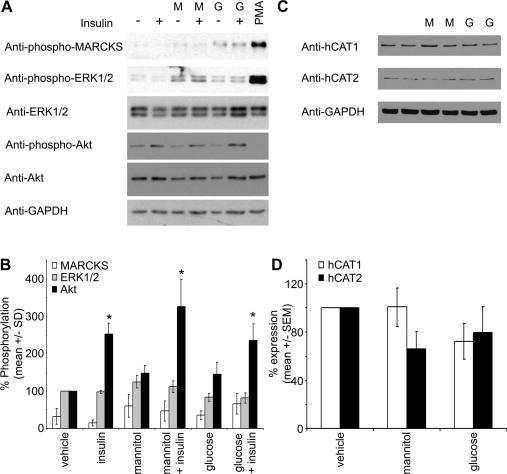
The effect of high glucose on ERK, Akt and PKC signalling in HAECs. Confluent HAECs were incubated in 5 mM glucose, 5 mM glucose + 20 mM mannitol (M) or 25 mM glucose (G) for 48 h and subsequently incubated in the presence or absence of insulin or PMA (1 μM, 10 min). Lysates were prepared and subjected to immunoblotting with the antibodies indicated. (A) and (C) Representative blots from six (ERK1/2, Akt), three (MARCKS) or five (hCAT1, hCAT2) independent experiments are shown. (B) Phosphorylation was quantified relative to total expression (GAPDH in the case of phospho-MARCKS) in each lysate. (D) Expression was quantified relative to GAPDH in each lysate.

To determine whether insulin-stimulated l-arginine transport was impaired by culture in high glucose, we cultured cells in 5 mM or 25 mM glucose for 48 h and assessed basal and insulin-stimulated l-arginine transport. High glucose significantly increased basal l-arginine transport 2.1 ± 0.4-fold (Fig. 2A). Culture glucose concentration had no significant effect on insulin-stimulated l-arginine transport, yet insulin elicited a significant 37% reduction in l-arginine transport in HAECs cultured in high glucose (Fig. 2A). The glucose-stimulated increase in basal l-arginine transport was unaffected by preincubation with wortmannin, but was abrogated by incubation with the MEK inhibitor, PD98059, or the PKC inhibitor, bisindolylmaleimide I (Fig. 2B). High glucose had no effect on basal or insulin-stimulated Akt Ser473 phosphorylation (Fig. 3). Neither high glucose nor insulin had any effect on the phosphorylation of either MARCKS or ERK1/2, except for a modest, non-significant increase in ERK 1/2 phosphorylation in response to the osmotic control, mannitol (Fig. 3). PMA elicited a marked increase in the phosphorylation of both MARCKS and ERK1/2 (Fig. 3). HAECs expressed both hCAT1 and hCAT2B, but not hCAT2A, as assessed by semi-quantitative PCR (data not shown). High glucose had no effect on the mRNA expression (data not shown) or protein levels of hCAT1 or hCAT2 (Fig. 3).

## 4 Discussion

The principal findings of this study are that insulin rapidly stimulates l-arginine transport, an effect attenuated by the PI3 K inhibitor, wortmannin, or infection with adenoviruses expressing dominant-negative mutant Akt. Furthermore, downregulation of l-arginine transport attenuates insulin-stimulated NO synthesis.

The relationship between arginine supply and nitric oxide synthesis is complex. Several reports have indicated that exogenous l-arginine improves NO synthesis, yet the intracellular concentrations of l-arginine exceed the K_M_ of eNOS, such that additional l-arginine should not further stimulate NO synthesis – the ‘arginine paradox’ [15,16]. Previous studies in HUVECs demonstrated that prolonged incubation with insulin stimulated l-arginine transport in a manner sensitive to the protein synthesis inhibitor cycloheximide [9] and concomitantly increased mRNA expression of CAT transporters, an effect abrogated by inhibitors of PI3K, PKC and ERK 1/2 [17]. The rapid stimulation by insulin reported in this study, however, is unlikely to reflect de novo synthesis of transporter proteins and was, at least in part, mediated by Akt activation, suggesting insulin-stimulated Akt activity directly phosphorylates transporters or stimulates transporter activity through a less direct mechanism. The observation that basal and insulin-stimulated HAEC NO synthesis was increased by extracellular l-arginine indicates that l-arginine transport may indeed contribute to insulin-stimulated NO synthesis. In support of this hypothesis, the effect was abrogated by co-incubation (for 5 min) with high concentrations of l-lysine, used in the l-arginine transport assay to inhibit transport. A similar effect has been reported in A23187-stimulated EA.hy926 cells by l-lysine [18].

Culture in high glucose concentrations markedly increased l-arginine transport in HAECs, in agreement with previous studies in HUVECs [9]. PCR experiments demonstrated that HAECs expressed mRNA for hCAT1 and hCAT2B, but not hCAT2A, as has previously been reported in HUVECs [19]. One previous study in HUVECs has reported that incubation in high glucose for 6 h markedly increased hCAT1 mRNA levels [20], yet the level of hCAT1 protein was not reported. In the current study, hCAT1 and hCAT2 protein levels in HAECs were unaffected by glucose culture concentration, indicating that the glucose-stimulated change in arginine transport is likely to reflect a change in activity, rather than expression of the hCAT isoforms. Furthermore, the effect of high glucose was attenuated by inhibitors of either ERK1/2 or PKC but was insensitive to wortmannin, suggesting that both PKC and ERK1/2 activity contribute to increased l-arginine transport. In the current study, PKC activity was not altered by glucose and/or insulin as assessed by phospho-MARCKS immunoblots. In addition, no change in ERK 1/2 phosphorylation or expression was observed in response to glucose and/or insulin. Incubation with high glucose has been reported to rapidly (5 min–6 h) stimulate PKC activity in HAECs and HUVECs and ERK phosphorylation in HUVECs [20–22]. The different effects of high glucose on PKC and ERK1/2 phosphorylation compared to these more short-term studies are likely to reflect the very different timescales of stimulation with high glucose. Indeed, in HAECs, prolonged high glucose culture conditions were reported to have no effect on ERK1/2 phosphorylation [23], in agreement with the current study.

Taken together, these data suggest that insulin rapidly stimulates l-arginine transport via a PI3K-Akt-dependent mechanism, which contributes to insulin-stimulated NO synthesis. High glucose conditions also increase l-arginine transport in an ERK1/2 and PKC inhibitor-sensitive manner. As we detected no apparent activation of either of these kinases under high glucose conditions, it seems likely that basal ERK1/2 and PKC activity are required for the response to high glucose, rather than activation itself. It is clear, therefore, that there is no simple quantitative relationship between arginine transport and NO synthesis, as insulin stimulates NO synthesis and l-arginine transport, but inhibits high glucose stimulated l-arginine transport, in agreement with previous studies in HUVECs [9]. Indeed, insulin-stimulated HAECs cultured in normal or high glucose conditions had very similar rates of l-arginine transport, as described previously [9], yet elicit different rates of NO synthesis [5]. It therefore is likely that although l-arginine transport contributes to insulin-stimulated NO synthesis in normal glucose concentrations, impaired l-arginine transport does not underlie the impaired insulin-stimulated NO synthesis observed in HAECs cultured in high glucose [5].

In conclusion, we propose that while increased supply of l-arginine contributes to insulin-stimulated NO synthesis, reduced insulin-stimulated l-arginine transport does not explain the specific reduction in insulin-stimulated NO synthesis we have previously demonstrated under high glucose conditions. Further work is necessary to identify the mechanisms impaired by high glucose culture conditions. Such studies should include an analysis of the regulation of subcellular localisation of eNOS and association of eNOS with caveolin-1 and/or Hsp90.
